# Autonomic-vascular dysregulation in CKD-associated hypertension: a narrative review with evidence hierarchy

**DOI:** 10.3389/fnins.2026.1808065

**Published:** 2026-04-29

**Authors:** Omar Z. Ameer

**Affiliations:** Department of Pharmaceutical Sciences, College of Pharmacy, Alfaisal University, Riyadh, Saudi Arabia

**Keywords:** autonomic nervous system, baroreflex, chronic kidney disease, hypertension, renal afferent signaling, sympathetic overactivity, vascular remodeling

## Abstract

Hypertension and chronic kidney disease frequently coexist and mutually accelerate cardiovascular and renal injury. This narrative review prioritizes direct human autonomic phenotyping (Level 1: microneurography, HRV/BRS), human vascular correlates (Level 2: PWV, FMD), and complementary preclinical evidence (Level 3) to elucidate autonomic-vascular mechanisms. Autonomic imbalance, characterized by sympathetic overactivity and reduced parasympathetic restraint, represents a key interface between neural control and vascular pathology in this setting. This narrative review synthesizes experimental and clinical evidence on how the autonomic nervous system shapes vascular function in hypertension and CKD. We outline physiological autonomic control of vascular tone (baroreflex pathways, central networks, brain–kidney communication), characteristic autonomic alterations in hypertension (elevated MSNA, impaired HRV/BRS), and their vascular consequences (endothelial dysfunction, arterial stiffness). We emphasize CKD-specific autonomic drivers (renal afferents, uremic toxins, inflammation) and their translation to exaggerated vascular injury and adverse BP phenotypes. Finally, we discuss pharmacological/device-based strategies targeting autonomic–vascular pathways, highlighting opportunities for neuromodulation, biomarker-guided risk stratification, and individualized treatment. By integrating multidisciplinary evidence, this review frames CKD hypertension as amplified autonomic–vascular injury and positions the autonomic nervous system as a promising therapeutic target.

## 1 Introduction

Hypertension is both a cause and a consequence of chronic kidney disease (CKD), and their coexistence markedly amplifies cardiovascular and renal risk. Hypertension accelerates renal injury, while CKD increases cardiovascular risk through interacting hemodynamic, neurohumoral, and structural mechanisms. A growing body of experimental and clinical evidence has implicated altered renal hemodynamics, endothelial dysfunction, arterial stiffness, and autonomic imbalance in this cardiorenal axis. In particular, sympathetic nervous system (SNS) hyperactivity, renin–angiotensin–aldosterone system (RAAS) activation, endothelial dysfunction, and vascular remodeling form an interconnected network that sustains hypertension in CKD (Ameer, 2022). Within this framework, autonomic regulation of vascular tone and structure serves as a dynamic interface between neural, humoral, and hemodynamic stressors, yet it remains under-recognized in routine clinical care (Penne et al., 2009; Salman, 2015; Lai et al., 2020). Autonomic control of vascular function therefore represents a central driver of blood pressure (BP) burden and cardiovascular risk in CKD, alongside sodium overload, RAAS overactivation, oxidative stress, and structural vascular change as key contributors to disease progression (Saxena et al., 2018).

**Figure 1:**
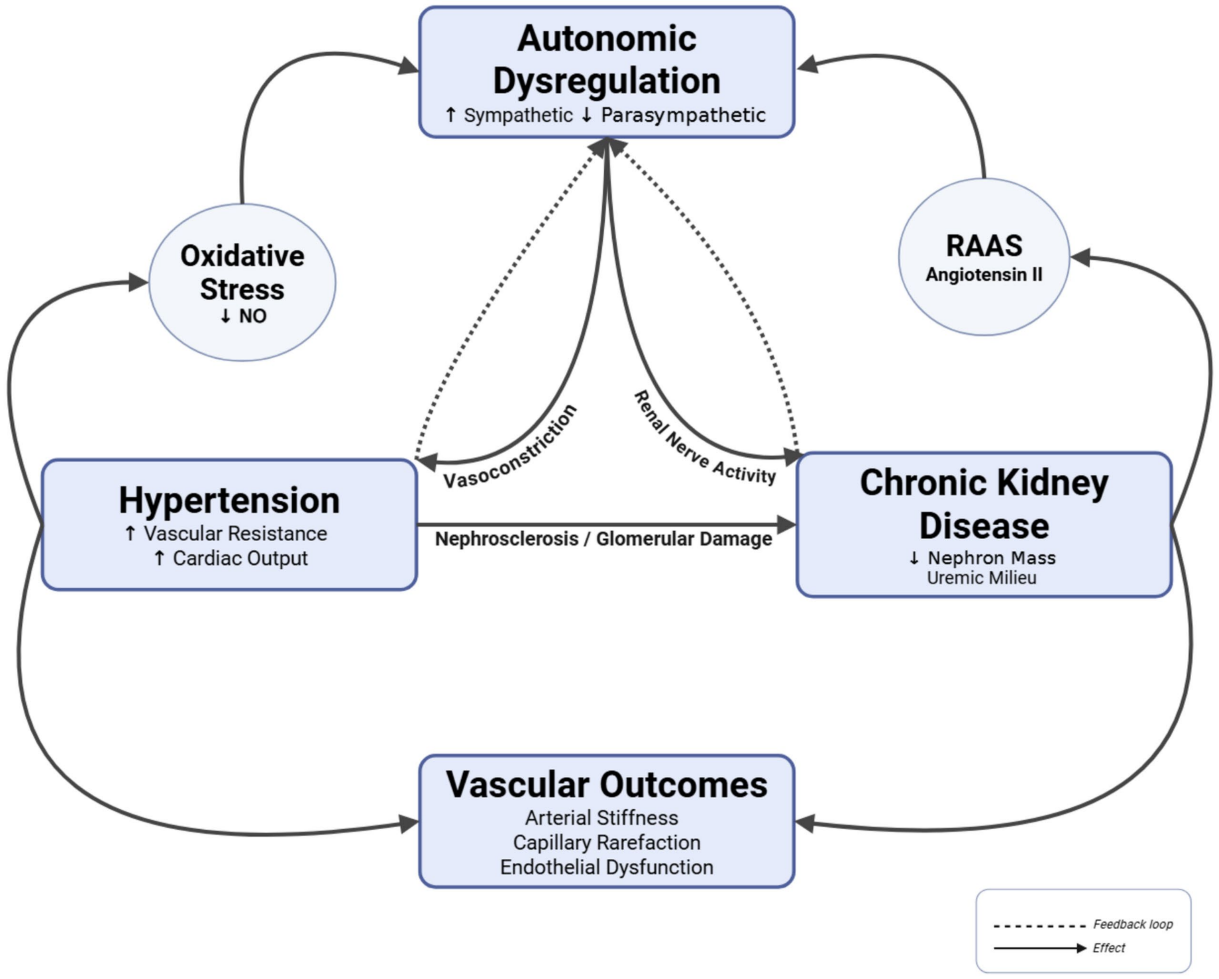
Autonomic dysregulation (↑sympathetic, ↓parasympathetic) drives hypertension via vasoconstriction and CKD via renal nerve activity. Central mechanisms: RAAS activation (angiotensin II), oxidative stress (↓NO bioavailability), and uremic milieu (↓nephron mass). Feedback loops perpetuate the cycle: hypertension → nephrosclerosis/glomerular damage → CKD → autonomic imbalance. Pathways converge on vascular outcomes (arterial stiffness, capillary rarefaction, endothelial dysfunction). Solid arrows, direct effects; dashed arrows, feedback loops. This figure is adapted and modified from Ameer (2022), Grassi et al. (2015), KenKnight and Verrier (2022) and made with BioRender.com software.

Autonomic imbalance, characterized by heightened sympathetic and reduced parasympathetic activity, emerges as a central mechanism linking hypertension, CKD, and their shared vascular complications. Increased sympathetic outflow and blunted vagal modulation promote systemic vasoconstriction and enhanced renal sympathetic nerve traffic, raising vascular resistance and cardiac output and thereby perpetuating hypertension, while also driving nephrosclerosis and progressive nephron loss (Esler, 2011; Mancia and Grassi, 2014) (Figure 1). Impaired cardiovagal baroreflex function further reduces buffering of beat-to-beat BP fluctuations and favors sympathetic dominance. In parallel, RAAS activation and oxidative stress amplify autonomic dysregulation, reduce nitric oxide (NO) bioavailability, and reinforce both hypertensive load and renal injury (Mancia and Grassi, 2014; Hall et al., 2024). The convergence of these processes on the vasculature leads to arterial stiffness, microvascular rarefaction, and endothelial dysfunction, which in turn worsen BP control and kidney damage, completing a feedforward brain–heart–kidney–vasculature loop that characterizes hypertension in CKD (Bidani et al., 2013; Mancia and Grassi, 2014) (Figure 1). Understanding how autonomic dysregulation shapes vascular function within this loop is crucial for developing targeted neuromodulatory and pharmacological strategies that complement standard antihypertensive and nephroprotective therapies (Grassi et al., 2015; Cheng et al., 2019; Quarti-Trevano et al., 2021; Seravalle et al., 2021; Kumar, 2025; Zoccali et al., 2025).

Therefore, the aim of this narrative review is to provide an integrated overview of how autonomic regulation of the vasculature contributes to the development and maintenance of hypertension in CKD, and how this, in turn, accelerates vascular and renal injury. Unlike prior reviews that treated these as parallel phenomena, our primary advance is threefold:

1. *Formal evidence hierarchy* (Level 1–3) systematically distinguishes human clinical data from preclinical mechanisms across all major claims.
2. *Direct human-clinical synthesis* juxtaposing microneurography (MSNA elevation), autonomic indices (HRV/BRS reduction), and vascular phenotyping (PWV, FMD impairment) in Tables 1–4.
3. *Translational treatment mapping* linking specific autonomic-vascular mechanisms to established (RAASi/SGLT2i) and emerging (RDN, BAT) therapies.

**Table 1:** Key autonomic abnormalities in essential hypertension.

Parameter	Direction vs. normotensive controls	Mechanistic implication	References
MSNA	Increased resting burst frequency and incidence	Enhanced vasoconstrictor drive; higher peripheral resistance; promotion of vascular remodeling	Mancia and Grassi (2014) and Grassi et al. (2015)
Plasma NE	Elevated baseline levels	Systemic marker of sympathetic overactivity; contributes to tachycardia and vasoconstriction	Grassi et al. (2015)
HRV – time and frequency domain indices	Reduced overall variability; decreased HF power; increased low-frequency/high-frequency (LF/HF) ratio	Depressed parasympathetic modulation; relative sympathetic predominance; impaired cardiac autonomic flexibility	Mancia and Grassi (2014)
Spontaneous baroreflex BRS	Decreased slope of RR interval–systolic BP relationship	Impaired buffering of acute BP changes; facilitates BPV and sustained sympathetic activation	Mancia and Grassi (2014) and Grassi et al. (2015)
Resting heart rate	Mildly increased	Reflects higher sympathetic and lower vagal tone; associated with greater cardiovascular risk	Mancia and Grassi (2014)
Arterial stiffness (e.g., carotid–femoral PWV)	Increased	Stiff arteries augment systolic load, widen PP, and contribute to baroreflex resetting toward higher pressures	Grassi et al. (2015)
Central sympathetic outflow to kidney (inferred from renal NE spillover)	Increased	Promotes renin release, sodium retention, and renal vasoconstriction, thereby sustaining hypertension	Mancia and Grassi (2014)

Key Autonomic and Vascular Abnormalities in Essential Hypertension (Evidence: Level 1 + 2). Characteristic pattern of direct autonomic dysfunction (Level 1: MSNA, HRV, BRS, NE) and vascular correlates (Level 2: PWV, heart rate) consistently observed across human hypertension studies, establishing the baseline neurovascular phenotype against which CKD-associated changes are compared. Note: Parameters reflect coherent shift toward sympathetic overactivity, parasympathetic withdrawal, and vascular stiffening in human essential hypertension. See Methods Section 2 for evidence hierarchy details. MSNA, muscle sympathetic nerve activity; NE, norepinephrine; HRV, heart rate variability; HF, high frequency; LF, low frequency; BRS, baroreflex sensitivity; BP, blood pressure; BPV, blood pressure variability; PP, pulse pressure; PWV, pulse wave velocity.

**Table 2:** Key human studies validating autonomic-vascular dysregulation in CKD (stages 2–5).

Study	CKD stage	Method	Key finding	Vascular correlate
Grassi et al. (2022)	2–4	MSNA	↑MSNA burst frequency	↑PWV, ↓FMD
Converse Jr et al. (1992)	Pre-dialysis	MSNA	Volume expansion ↑MSNA	N/A
Chandra et al. (2011)	3–5	HRV	↓HRV predicts CV events	CKD progression
Sabino-Carvalho et al. (2023)	3–5	BPV/cBRS	Blunted BRS → ↑BPV	Non-dipping BP
Sabino-Carvalho et al. (2024)	3–4	MSNA/BRS	Blunted sBRS + ↓sympathetic transduction	Counter-regulatory
Jeong et al. (2023)	3–4	MSNA/ AIx	Exercise prevents ↑MSNA and ↑AIx	↓Augmentation index

Key Human Studies Providing Evidence for Autonomic-Vascular Dysregulation in CKD (Stages 2–5; Evidence: Level 1 + 2). Summary of 6 key human studies demonstrating direct relationships between autonomic dysfunction (Level 1: MSNA, HRV, BRS) and vascular correlates (Level 2: PWV, FMD, BPV, AIx) across CKD stages 2–5, establishing translational evidence for neurovascular mechanisms. Studies selected based on direct Level 1 + 2 human phenotyping with vascular correlates. See Methods Section 2 for evidence hierarchy details. MSNA, muscle sympathetic nerve activity; PWV, pulse wave velocity; FMD, flow-mediated dilation; BRS, baroreflex sensitivity; HRV, heart rate variability; BPV, blood pressure variability; sBRS, sympathetic; cBRS, cardiac BRS; AIx, augmentation index.

**Table 3:** Clinical autonomic findings in chronic kidney disease and their relationships with blood pressure, vascular markers, and outcomes.

Study population (example cohorts)	Evidence type	Autonomic measures assessed	Main findings vs. matched controls	Associations with BP and vascular markers	Associations with clinical outcomes	Key outcomes
Non-dialysis CKD stages 3–5 (mixed etiologies), cross-sectional	Cohort (cross-sectional)	HRV (time and frequency domain), BRS	Lower overall HRV, reduced high-frequency power, and blunted BRS indicating autonomic imbalance.	Higher office and ambulatory BP, greater BPV, and higher carotid–femoral PWV in patients with lower HRV/BRS.	Reduced HRV and BRS linked to higher risk of LV and composite cardiovascular events over follow-up.	Level 1 + 2 (Sinha and Agarwal, 2015; Lai et al., 2020; Serhiyenko et al., 2023)
Hemodialysis patients (maintenance, thrice weekly)	Cohort (longitudinal)	HRV, BRS, post-dialysis autonomic tests	Markedly depressed HRV and baroreflex indices; dialysis sessions cause transient shifts, but baseline autonomic dysfunction persists.	Non-dipping or reverse-dipping nocturnal BP patterns and increased arterial stiffness associated with lower HRV indices.	Low HRV (especially reduced SDNN and HF power) predicts sudden cardiac death and all-cause mortality.	Level 1 + 2 (Kuo et al., 2018; Osataphan et al., 2023)
CKD stages 3–4 with and without diabetes	Case–control	HRV, orthostatic BP response	CKD groups show reduced HRV; diabetic CKD patients exhibit more pronounced autonomic neuropathy and orthostatic hypotension.	Autonomic dysfunction associates with higher nighttime BP load and impaired nocturnal dipping.	Presence of cardiovascular autonomic neuropathy associated with higher rates of circulatory events and hospitalizations.	Level 1 + 2 (Tahrani et al., 2014)
CKD (stages 2–4) vs. healthy controls, microneurography sub-study	Mechanistic sub-study	MSNA, HRV	CKD patients have significantly higher MSNA burst frequency and lower HRV compared with controls, indicating sympathetic overactivity.	Higher MSNA correlates with reduced systolic BP dipping, higher nighttime BP, and worse endothelial function.	Elevated MSNA linked indirectly to greater arterial stiffness and predicted cardiovascular risk in longitudinal analyses.	Level 1 + 2 (Kaur et al., 2017; Grassi et al., 2021)
CKD cohorts with autonomic and inflammatory profiling	Prognostic cohort	HRV, baroreflex indices, inflammatory markers	Autonomic dysfunction (low HRV, impaired BRS) clusters with higher inflammatory markers and oxidative stress indices.	Patients with combined autonomic dysfunction and elevated inflammation show higher central BP and PWV.	Synergistic autonomic–inflammatory abnormalities associate with faster CKD progression and higher circulatory event rates.	Level 1 + 2 (Zoccali et al., 2025)

Clinical Evidence of Autonomic Dysfunction Across CKD Stages (Evidence: Level 1 + 2). Summary of cohort-level findings linking reduced HRV, impaired BRS, and elevated MSNA to vascular injury, abnormal BP patterns, and adverse cardiovascular outcomes in CKD stages 3–5 and dialysis populations. Each row identifies evidence type and key prognostic associations. Evidence classification: Level 1 = direct human autonomic measures; Level 2 = human vascular correlates. See Methods for details. CKD, chronic kidney disease; HRV, heart rate variability; SDNN, standard deviation of normal-to-normal intervals; BRS, baroreflex sensitivity; BP, blood pressure; BPV, blood pressure variability; PWV, pulse wave velocity; LVH, left ventricle hypertrophy; HF, high frequency; vs, versus; MSNA, muscle sympathetic nerve activity.

**Table 4:** Autonomic-targeted interventions: evidence levels for CKD hypertension (level 1 + 2 vs. 2 + 3).

Intervention/strategy	Population (with CKD relevance)	Autonomic endpoints reported	Vascular/BP endpoints reported	Key outcomes and considerations for CKD patients	Evidence level
Intensive RAAS blockade: angiotensin converting enzyme inhibitors/ angiotensin AT1 receptor blockers ± mineralocorticoid receptor antagonists (ACEI/ARB ± MRA)	Hypertensive CKD (proteinuric and non-proteinuric)	Indirect sympatholytic effects via reduced angiotensin II; some studies show improved HRV and baroreflex indices with optimized RAAS inhibition.	Lower BP, reduced proteinuria, slower decline in eGFR; improved endothelial function and partial regression of LVH.	Cornerstone of CKD hypertension management; hyperkalemia and GFR decline require close monitoring, especially when MRAs are added.	Level 1 + 2
SGLT2 inhibitors	CKD with diabetes and/or heart failure; increasing data in non-diabetic CKD	Indirect autonomic effects via improved volume control, reduced renal tubular oxygen demand, and possibly reduced sympathetic drive; HRV data emerging.	BP lowering (typically 3–5 mmHg systolic), reduced arterial stiffness and improved central hemodynamics in some studies; strong renal and heart failure outcome benefits.	Very attractive option in CKD patients within eGFR label; monitor for volume depletion and rare ketoacidosis; autonomic benefits are likely contributory but not the primary indication.	Level 1 + 2
Renal sympathetic denervation – catheter-based radiofrequency or ultrasound	Resistant hypertension (subsets including CKD stages 3–4 in several renal denervation trials and registries)	Indirect: reductions in sympathetic drive inferred from decreased BP, improved HRV in some cohorts; direct MSNA reduction shown in small mechanistic studies.	Sustained office and ambulatory BP reductions (5–10 mmHg systolic in more recent sham-controlled trials); modest improvements in arterial stiffness and central BP in responders.	Promising option in resistant hypertension with CKD stages 3–4; requires preserved renal artery anatomy and carries procedural risks; long-term renal safety acceptable in carefully selected patients.	Level 2 + 3
BAT – implantable carotid stimulator	Severe resistant hypertension, many with CKD or reduced eGFR at baseline	Improved BRS, reduced MSNA in mechanistic sub-studies; increased HRV indices indicating better autonomic balance.	Meaningful and sustained reductions in systolic BP (often ≥15–20 mmHg in early studies); reductions in central BP and some improvement in arterial stiffness indices.	Invasive device requiring surgery; CKD patients may benefit from BP reduction, but careful selection and monitoring are needed due to infection and procedural risks.	Level 2 + 3
Vagus nerve stimulation – implantable or external	Chronic heart failure and high-risk cardiovascular patients, including individuals with CKD or reduced eGFR in heart-failure cohorts	Increased HRV, improved vagal indices, reduced sympathetic surrogates, and lower circulating inflammatory markers.	Improved functional class and, in some studies, better LV function; BP changes modest and variable, but central hemodynamics and arrhythmia burden may improve.	Could be attractive in CKD patients with heart failure and high sympathetic tone; data specifically in CKD are limited, and device implantation adds complexity.	Level 2 + 3
Lifestyle and exercise-based autonomic rehabilitation (aerobic training, controlled breathing)	CKD stages 3–5 and dialysis populations in small randomized controlled trials s and pilot studies	Improved HRV (higher HF power, lower LF/HF), reduced resting heart rate, and better BRS.	Modest BP reductions, improved endothelial function, and decreased arterial stiffness indices in responders.	Low-cost, non-pharmacologic approach; adherence and exercise capacity may be limiting; should be integrated with medical therapy for CKD hypertension.	Level 2

Autonomic-Targeted Interventions: Evidence Levels for CKD Hypertension (Level 1 + 2 vs 2 + 3). Comparative stratification distinguishing established therapies with proven CKD outcomes and direct/indirect autonomic effects (Level 1 + 2: RAAS blockade, SGLT2i) from emerging strategies supported primarily by surrogate autonomic endpoints or extrapolated CKD data (Level 2 + 3: device therapies; Level 2: exercise). Evidence classification: Level 1 = direct human autonomic measures (microneurography, HRV/BRS); Level 2 = human vascular correlates/surrogate endpoints (PWV, FMD, BP patterns); Level 3 = preclinical models or mechanistic studies. See Methods Section 2 for details. RAAS = renin–angiotensin–aldosterone system; AT1, angiotensin receptor type 1; ACEI, angiotensin converting enzyme inhibitor; ARB, angiotensin receptor blocker; MRA, mineralocorticoid receptor antagonist; CKD, chronic kidney disease; BP, blood pressure; eGFR, estimated Glomerular Filtration Rate; LVH, left ventricle hypertrophy; SGLT2, sodium–glucose co-transporter 2; HRV, heart rate variability; MSNA, muscle sympathetic nerve activity; mmHg, millimeters of mercury; BAT, Baroreflex activation therapy; BRS, baroreflex sensitivity; BP, blood pressure; CKD, chronic kidney disease; LV, left ventricle; HF, high frequency; LF, low frequency.

This unified framework provides evidence that CKD hypertension represents a distinct neurovascular phenotype rather than simply “more severe” essential hypertension.

This review applies this framework to distinguish associative clinical findings from more strongly supported causal mechanisms and clearly separate mechanistic insights from experimental CKD models from direct human evidence. Where translation remains uncertain, we highlight these gaps rather than inferring causality. This review is structured around a clear evidence hierarchy: Level 1 = direct human autonomic measures (microneurography, HRV/BRS); Level 2 = human vascular correlates (PWV, FMD, ambulatory patterns); Level 3 = preclinical CKD models. Each major claim is flagged at section start (e.g., “Evidence: Level 1 + 2”) and critically appraised for associative vs. causal support. This distinguishes robust human findings from model-based mechanistic hypotheses, preventing overinterpretation.

## 2 Literature search and synthesis approach

This review was conducted as a narrative, hypothesis-driven synthesis of the literature on autonomic regulation of vascular function in hypertension and CKD. We searched PubMed/MEDLINE, Embase, and Scopus for articles published up to March 2026 using combinations of terms related to autonomic function, including “sympathetic nerve activity,” “baroreflex,” and “heart rate variability,” together with terms for hypertension and CKD. Reference lists of key reviews and original articles were also screened to identify additional relevant studies.

We prioritized peer-reviewed original research and systematic reviews that provided direct autonomic or vascular phenotyping in humans, such as microneurography, heart rate variability (HRV), baroreflex testing, carotid–femoral pulse wave velocity, and flow-mediated dilation. Preclinical work in established CKD models was included when it offered essential mechanistic insight, particularly for pathways that cannot be directly interrogated in humans. Because the evidence base is heterogeneous, no formal meta-analysis was performed; instead, findings were synthesized qualitatively.

To aid interpretation, we conceptually organized the evidence into three levels: Level 1, direct human autonomic measures; Level 2, human vascular correlates; and Level 3, preclinical CKD models. This framework was used throughout the review to distinguish associative clinical observations from more strongly supported mechanistic evidence.

## 3 Physiological autonomic control of vascular function

### 3.1 Sympathetic and parasympathetic influences

**Figure 2:**
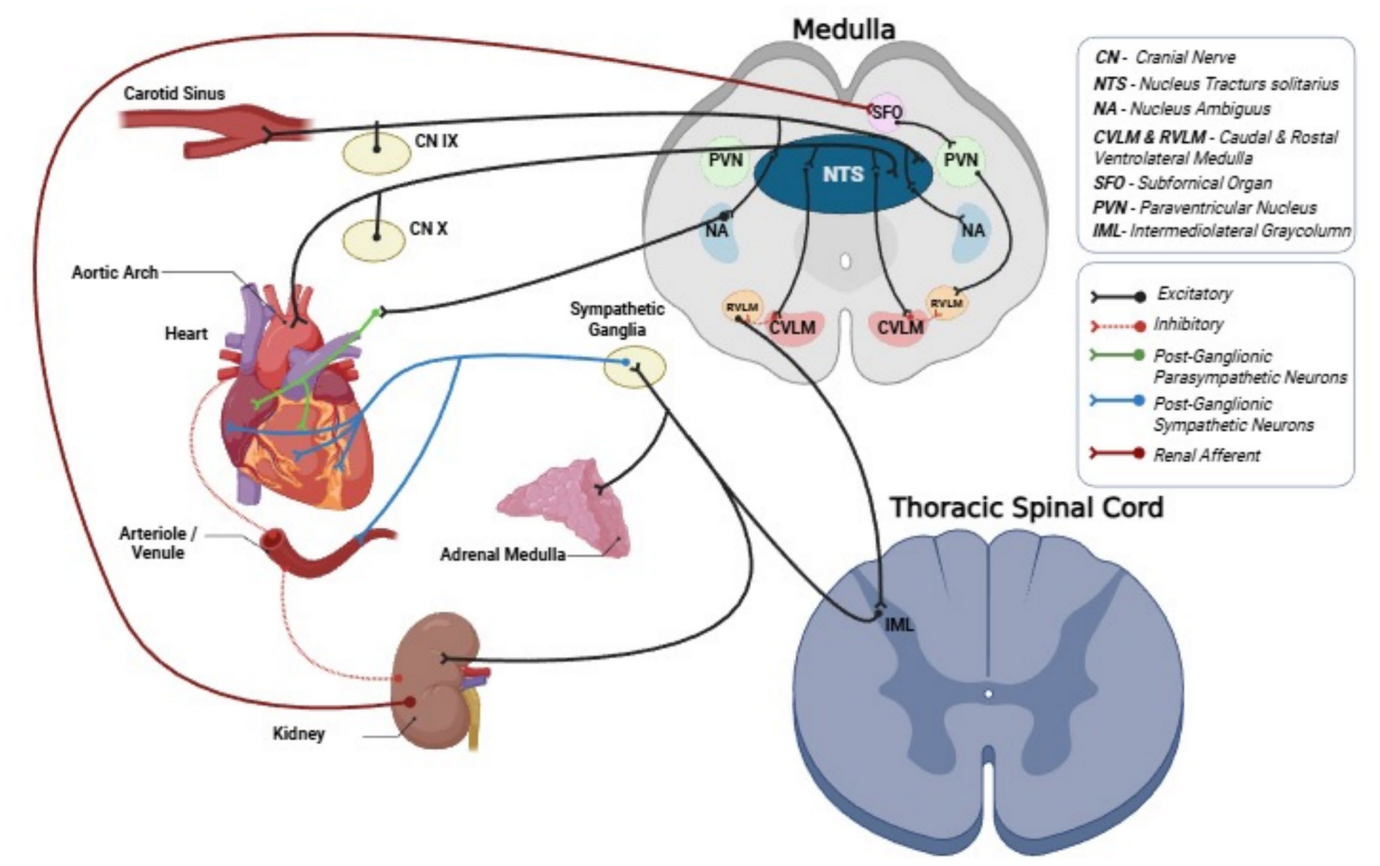
Central integration of the arterial baroreflex, cardiac and renal sympathetic innervation. Sinoaortic baroreceptors located in the carotid sinus and aortic arch send afferent signals via cranial nerves IX and X to the nucleus tractus solitarius (NTS) in the dorsal medulla, where information on beat-to-beat arterial pressure is integrated. The NTS projects to the caudal and rostral ventrolateral medulla (CVLM, RVLM), nucleus ambiguus (NA), and hypothalamic nuclei including the paraventricular nucleus (PVN), with modulatory input from the subfornical organ (SFO). Excitatory and inhibitory connections within this network regulate sympathetic premotor neurons in the RVLM, which descend to preganglionic sympathetic neurons in the intermediolateral cell column (IML) of the thoracic spinal cord. From the IML, efferent fibers project via sympathetic ganglia to the heart, resistance vessels, adrenal medulla, and kidney. Parasympathetic efferents arising from the NA travel with the vagus nerve to the heart, whereas renal afferent nerves convey information from the kidney back to the brainstem and hypothalamus, completing a bidirectional brain–heart–kidney axis. CVLM, caudal ventrolateral medulla; IML, intermediolateral cell column; NTS, nucleus tractus solitarius; PVN, paraventricular nucleus; RVLM, rostral ventrolateral medulla; SFO, subfornical organ. This figure is adapted and modified from Kaur et al. (2016), Katsurada and Patel (2024), and Dampney et al., 2002 and made with BioRender.com software.

Sympathetic efferent fibers exert direct control over arteriolar tone, venous capacitance, and renal hemodynamics by releasing norepinephrine (NE) onto vascular smooth muscle and the juxtaglomerular apparatus (DiBona, 1985; Sata et al., 2018), thereby influencing systemic vascular resistance, venous return, and renin release. On the other hand, parasympathetic efferent activity, primarily via the vagus nerve, exerts its cardiovascular effects through the sinoatrial and atrioventricular nodes (Purves et al., 2001), reducing heart rate and altering cardiac output (Klabunde, 2011; Hall and Hall, 2020); these cardiac changes, in turn, influence arterial pressure, pulse pressure (PP), and endothelial shear stress, with secondary consequences for vascular function. Thus, the autonomic nervous system regulates vascular function via sympathetic efferents that release NE onto vascular smooth muscle and parasympathetic influences that act largely indirectly through heart rate, cardiac output, and vascular mediators (Figure 2). In contrast to the SNS, which exerts direct vasoconstrictor effects via perivascular nerve terminals, parasympathetic influences on vascular tone are mainly indirect, operating through changes in heart rate and cardiac output, as well as through modulation of endothelial function and circulating vasodilators (for example, NO and other endothelium-derived relaxing factors) (Xavier, 2020). Consequently, parasympathetic activation primarily reduces arterial pressure by slowing heart rate and augmenting diastolic filling, with secondary consequences for shear stress and endothelial mediator release rather than via direct vasodilation at most resistance vessels.

Furthermore, baroreceptor and volume-sensing reflexes integrate these sympathetic and parasympathetic influences in response to pressure changes to stabilize arterial pressure on a beat-to-beat basis (Hall and Hall, 2020), and alterations in baroreflex sensitivity and operating point have been documented in hypertension (Narkiewicz and Grassi, 2008) and CKD (Chesterton and McIntyre, 2005). Under physiological conditions, changes in arterial pressure are buffered by the arterial baroreflex, which modulates sympathetic vasoconstrictor outflow and heart rate, while NO and other vasodilators further limit excessive increases in vascular tone and BP (Mancia and Grassi, 2014; Hall and Hall, 2020; Ameer, 2022). Afferent renal and cardiopulmonary mechanoreceptors additionally modulate central autonomic networks, influencing systemic vascular resistance and volume regulation (Mancia and Grassi, 2014; Salman et al., 2014; Kopp, 2015; Ameer, 2022).

*Evidence summary:* Sympathetic efferents have direct vascular and renal effects, whereas parasympathetic influences on vascular function are mainly indirect through heart rate, cardiac output, and endothelial modulation.

### 3.2 Central integrative networks

Central autonomic control involves the nucleus tractus solitarius (NTS), rostral ventrolateral medulla (RVLM), hypothalamus, and higher centers that integrate inputs from baroreceptors, chemoreceptors, and humoral signals such as angiotensin II and cytokines (Salman, 2016; Guyenet et al., 2020; Katsurada and Patel, 2024). This central network finely tunes vascular tone, heart rate, and renin release, coordinating long-term BP regulation (Figure 2) (Salman, 2015; Kaur et al., 2016; Maranduca et al., 2023). In healthy individuals, heart rate variability (HRV) and baroreflex sensitivity (BRS) reflect a balanced sympathetic–parasympathetic interplay, and changes in these indices can serve as early markers of autonomic dysregulation (Salman, 2015; Salman et al., 2015a; Kaur et al., 2016).

Baroreceptors are stretch-sensitive endings in both high-pressure (carotid sinus, aortic arch) and low-pressure regions of the circulation (Salman, 2016). High-pressure sinoaortic baroreceptors dominate short-term, beat-to-beat BP regulation, and impaired baroreflex function contributes to disorders with excessive blood pressure variability (BPV), especially during postural change. Sinoaortic afferents lie in the adventitia of the carotid sinus and aortic arch and fire in phase with the arterial pressure waveform, encoding both its level and rate of rise. Their signals travel via the glossopharyngeal nerve (carotid sinus) and vagus nerve (aortic arch) to the NTS in the dorsal medulla, which represents the central integration site of the baroreflex arc. Within the medulla, the NTS projects to caudal and rostral ventrolateral medulla (CVLM, RVLM) and to the nucleus ambiguous (Katsurada and Patel, 2024). The CVLM and RVLM shape sympathetic outflow to preganglionic neurons in the intermediolateral cell column of the thoracic spinal cord, which then project to sympathetic ganglia, resistance vessels, the heart, and the adrenal medulla (Esler, 2011; Guyenet et al., 2020). Neurons in the nucleus ambiguus give rise to preganglionic parasympathetic fibers in the vagus that innervate the sinoatrial node and other cardiac structures (Figure 2) (Guyenet et al., 2020). An increase in arterial pressure enhances baroreceptor firing, which drives NTS activation. This, in turn, boosts cardiovagal efferent activity and suppresses sympathetic efferents to the heart and vasculature, slowing heart rate and reducing contractility, peripheral resistance, and venous return (Esler, 2011). The resulting fall in pressure toward its operating set point exemplifies negative-feedback control through two opposing arms: a rapid vagal limb (latency hundreds of milliseconds) and a slower sympathetic limb (latency several seconds), reflecting faster cholinergic versus slower adrenergic signaling at target tissues. The kidney is richly innervated by efferent sympathetic fibers and by sensory afferent fibers that link renal status to central autonomic circuits (Katsurada and Patel, 2024). Renal sympathetic efferents, arising from preganglionic neurons in the thoracic and upper lumbar intermediolateral cell column and relayed through sympathetic ganglia, regulate renal blood flow, renin release, and tubular sodium reabsorption (Katsurada and Patel, 2024). In parallel, renal afferent nerves transmit chemical and mechanical signals from the kidney to the NTS, PVN, and other brainstem–hypothalamic nuclei, where they modulate global sympathetic tone and baroreflex function (Figure 2). This bidirectional neural communication means that renal injury and altered renal hemodynamics can drive sympathetic overactivity (Ameer et al., 2014; Salman et al., 2014; Salman et al., 2015a; Salman et al., 2015b), while heightened sympathetic drive further aggravates renal vasoconstriction, sodium retention, and hypertension (Saxena et al., 2018; Hall et al., 2024).

*Evidence summary:* Human physiology directly supports baroreflex and renal afferent control of autonomic balance, but the finer central mechanisms are more completely defined in experimental and translational studies.

## 4 Autonomic dysregulation in hypertension

Table 1 gives a summarized characteristic pattern of autonomic and vascular abnormalities observed in human hypertension from different robust studies, to highlight the coherent shift toward heightened sympathetic drive and impaired vagal and baroreflex buffering. Thus, the measurement of muscle sympathetic nerve activity (MSNA) and plasma NE levels are evidently consistent (Seravalle et al., 2021), reflecting augmented vasoconstrictor tone, increased peripheral resistance, and promotion of vascular remodeling (Ameer et al., 2015), while central sympathetic outflow to the kidney promotes renin release, sodium retention, and renal vasoconstriction, contributing to BP elevation (Mancia and Grassi, 2014; Grassi et al., 2015; Saxena et al., 2018; Hall et al., 2024). In parallel, reduced HRV, a higher low frequency/ high frequency (LF/HF) ratio, and blunted spontaneous BRS indicate depressed parasympathetic modulation and impaired feedback control of beat-to-beat pressure fluctuations, changes that contribute to BPV and long-term cardiovascular risk (Mancia and Grassi, 2014; Serhiyenko et al., 2023). These autonomic disturbances coexist with mild tachycardia and increased arterial stiffness, often assessed by carotid–femoral pulse wave velocity (PWV), which further augment systolic load, widen PP, and may reset baroreflex function toward higher operating pressures (Grassi et al., 2015; Serhiyenko et al., 2023). Together, the parameters in this table underscore that hypertension is not merely a hemodynamic disorder but also manifests as a systemic state of autonomic imbalance with direct consequences for vascular and renal structure and function (Mancia and Grassi, 2014; Grassi et al., 2015; Seravalle et al., 2021).

*Evidence summary:* In humans, reduced HRV, impaired baroreflex sensitivity, and elevated sympathetic activity are established markers of autonomic imbalance in hypertension, while the downstream vascular consequences are supported by clinical correlates and experimental data.

### 4.1 Sympathetic overactivity and blunted parasympathetic tone

**Figure 3:**
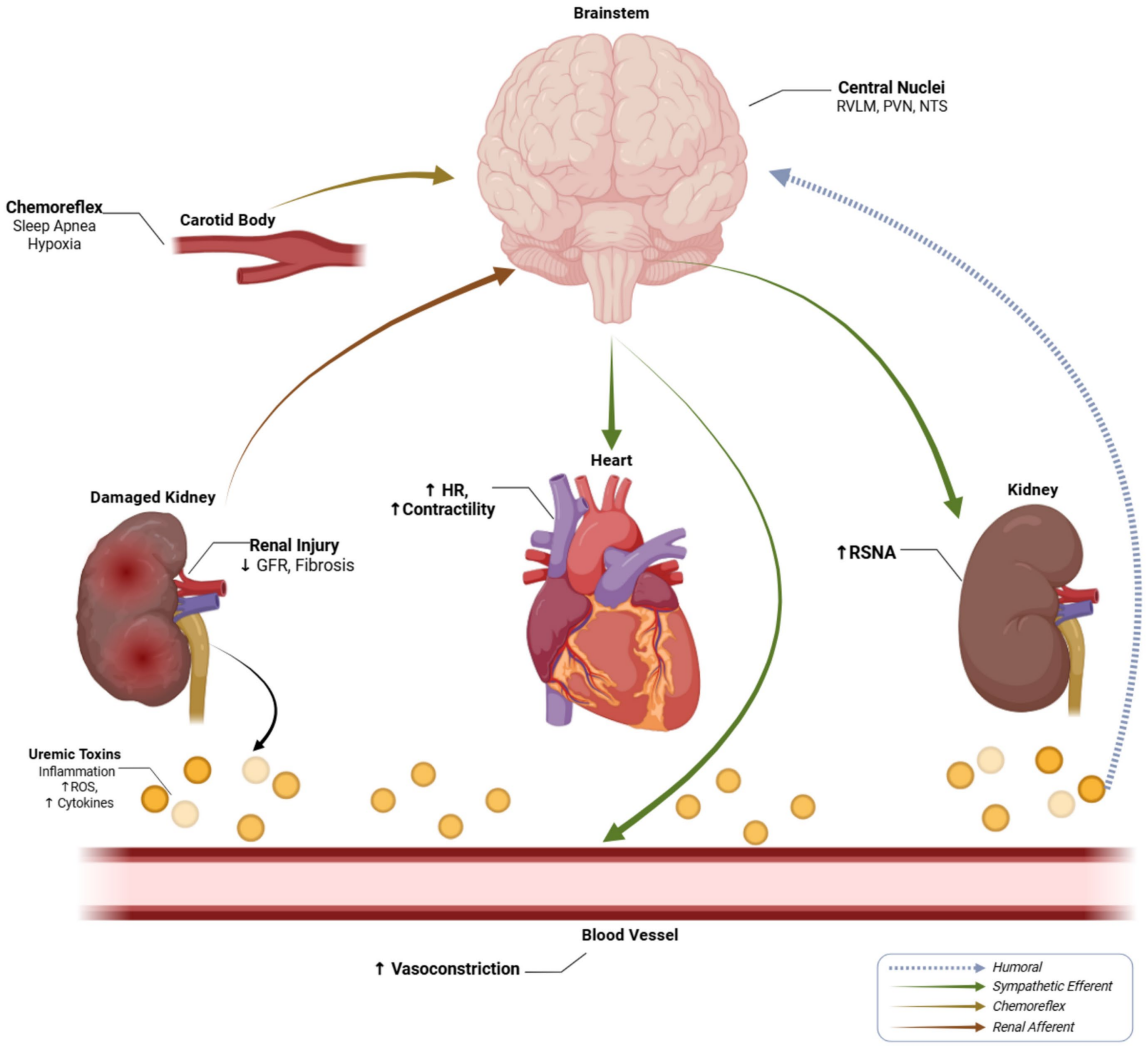
Mechanisms of sympathetic overactivity in chronic kidney disease. Renal injury activates afferent sensory nerves (red) projecting to central autonomic nuclei (RVLM, PVN, NTS). Circulating uremic toxins and inflammatory mediators (blue) released from the damaged kidney directly stimulate central nuclei via the humoral pathway. Chemoreflex sensitization from sleep apnea and intermittent hypoxia (yellow) provides additional excitatory input via the carotid body. Increased central sympathetic efferent outflow (green) targets the heart (↑HR, ↑contractility), kidneys (↑RSNA), and vasculature (↑vasoconstriction), perpetuating hypertension and CKD progression. This figure is adapted and modified from Ameer (2022), Grassi et al. (2015), and KenKnight and Verrier (2022) and made with BioRender.com software.

**Figure 5:**
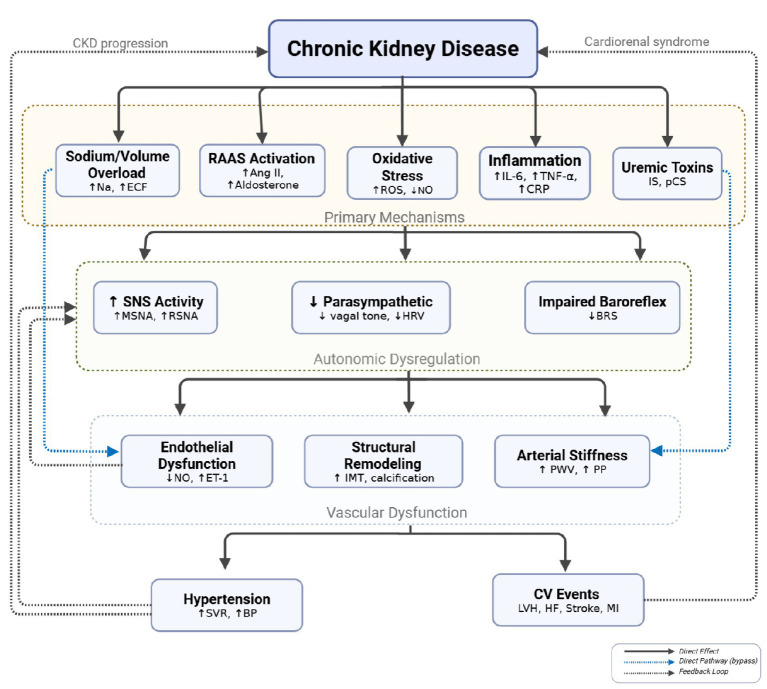
Integrated pathophysiology of CKD-associated hypertension. CKD initiates multiple primary mechanisms including sodium/volume overload (↑Na, ↑ECF), RAAS activation (↑Ang II, ↑aldosterone), oxidative stress (↑ROS, ↓NO), inflammation (↑IL-6, ↑TNF-α, ↑CRP), and uremic toxin accumulation (IS, pCS). These converge on autonomic dysregulation characterized by ↑SNS activity (↑MSNA, ↑RSNA), ↓parasympathetic tone (↓vagal tone, ↓HRV), and impaired baroreflex (↓BRS). Autonomic imbalance, along with direct pathways (blue dashed), drives vascular dysfunction: endothelial dysfunction (↓NO, ↑ET-1), structural remodeling (↑IMT, calcification), and arterial stiffness (↑PWV, ↑PP). These culminate in hypertension (↑SVR, ↑BP) and CV events (LVH, HF, stroke, MI). Feedback loops perpetuate CKD progression and cardiorenal syndrome. Ang II, angiotensin II; BP, blood pressure; BRS, baroreflex sensitivity; CKD, chronic kidney disease; CRP, C-reactive protein; CV, cardiovascular; ECF, extracellular fluid; ET-1, endothelin-1; HF, heart failure; HRV, heart rate variability; IL-6, interleukin-6; IMT, intima-media thickness; IS, indoxyl sulfate; LVH, left ventricular hypertrophy; MI, myocardial infarction; MSNA, muscle sympathetic nerve activity; NO, nitric oxide; pCS, p-cresyl sulfate; PP, pulse pressure; PWV, pulse wave velocity; RAAS, renin-angiotensin-aldosterone system; ROS, reactive oxygen species; RSNA, renal sympathetic nerve activity; SNS, sympathetic nervous system; SVR, systemic vascular resistance; TNF-α, tumor necrosis factor-alpha. This figure is adapted and modified from Ameer (2022), Grassi et al. (2015), Guyenet et al. (2020) and made with BioRender.com software.

Essential hypertension is characterized by increased sympathetic outflow, especially to the heart, kidney, and skeletal muscle vasculature, alongside impaired vagal modulation (Figure 3). Microneurography and NE spillover studies demonstrate elevated MSNA, which correlates with BP levels and target organ damage (Figure 5) (Mancia and Grassi, 2014; Grassi et al., 2015; Seravalle et al., 2021). Reduced HRV and depressed BRS provide non-invasive evidence of autonomic imbalance in hypertensive patients. These changes contribute not only to elevated BP but also to arrhythmogenesis and adverse cardiovascular outcomes (Quarti-Trevano et al., 2021; KenKnight and Verrier, 2022; Serhiyenko et al., 2023). However, most human data in essential hypertension are observational, and interventions that selectively normalize sympathetic indices without concurrent changes in BP or volume status remain limited, so definitive proof that correcting autonomic imbalance independently improves hard outcomes is still lacking.

*Evidence summary:* Most human data in essential hypertension are observational, so autonomic correction as an independent pathway to improved hard outcomes remains insufficiently proven.

### 4.2 Impact on vascular structure and function

**Figure 4:**
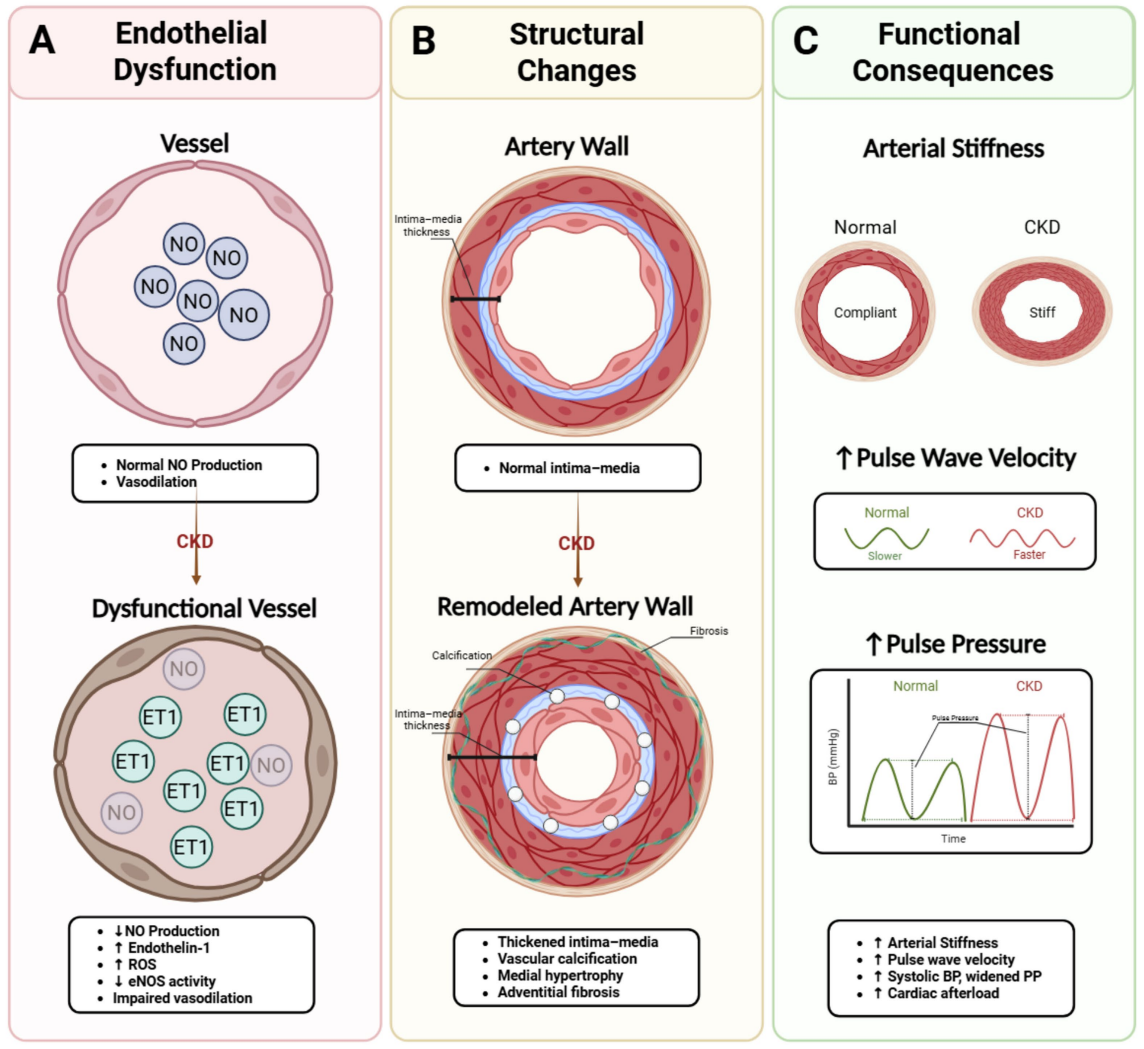
Vascular changes in CKD-associated hypertension. **(A)** Endothelial dysfunction: CKD reduces nitric oxide (NO) bioavailability and increases endothelin-1 (ET-1), reactive oxygen species (ROS), and impairs eNOS activity, leading to impaired vasodilation. **(B)** Structural changes: arterial remodeling includes intima-media thickening, vascular calcification, medial hypertrophy, and adventitial fibrosis. **(C)** Functional consequences: increased arterial stiffness elevates pulse wave velocity (PWV) and widens pulse pressure (PP), increasing systolic BP and cardiac afterload. BP, blood pressure; CKD, chronic kidney disease; eNOS, endothelial nitric oxide synthase; ET-1, endothelin-1; NO, nitric oxide; PP, pulse pressure; PWV, pulse wave velocity; ROS, reactive oxygen species. This figure is adapted and modified from Ameer (2022), Grassi et al. (2015), KenKnight and Verrier (2022), Mancia and Grassi (2014), and Roumeliotis et al. (2020) and made with BioRender.com software.

Chronic sympathetic overactivity promotes vasoconstriction, vascular smooth muscle hypertrophy, and increased collagen deposition, fostering arterial stiffness and structural remodeling (Figure 4). Angiotensin II and aldosterone, often upregulated in hypertension, synergize with SNS activity to enhance oxidative stress, reduce NO bioavailability, and worsen endothelial dysfunction (Mancia and Grassi, 2014; Ameer, 2022; Maranduca et al., 2023). Arterial stiffness, reflected by elevated PWV, further loads baroreceptors and may contribute to baroreflex resetting, reinforcing sympathetic dominance in a vicious cycle. These processes move the vasculature from a responsive, compliant state toward a rigid, high-resistance configuration typical of longstanding hypertension (Mancia and Grassi, 2014; Ameer, 2022; KenKnight and Verrier, 2022; Hall et al., 2024).

*Evidence summary:* Chronic sympathetic overactivity and RAAS activation are consistently linked to arterial stiffness and endothelial dysfunction, but the causal sequence is best established in experimental studies.

## 5 Chronic kidney disease as a driver of autonomic imbalance

### 5.1 Renal afferent signaling and central activation (evidence: level 1 + 2 + 3)

Both experimental CKD models (including Lewis Polycystic Kidney (LPK) rat) and direct human microneurography studies demonstrate that CKD serves as a potent stimulus for sympathetic activation, with increased sympathetic nerve traffic and NE spillover observed in reduced renal mass states. In LPK rats and other preclinical models, afferent signals from diseased kidneys including mechanosensory and chemosensory inputs activated by renal ischemia, interstitial inflammation, and fibrosis reach brainstem and hypothalamic centers, augmenting central sympathetic outflow (Figure 3) (Ameer et al., 2014; Salman et al., 2014). These preclinical findings are corroborated by human evidence: renal denervation attenuates afferent-driven sympathetic overactivity in experimental models (Veiga et al., 2020; Li et al., 2024), while microneurographic studies confirm elevated muscle sympathetic nerve activity (MSNA) in human CKD stages 2–5 (Converse et al., 1992; Grassi et al., 2021; Jeong et al., 2023). Convergent preclinical and clinical data show these signals contribute to systemic vasoconstriction, elevated renin release, and heightened BPV (Grassi et al., 2010; Johns and Abdulla, 2013; Mancia and Grassi, 2014). Taken together, animal and human data strongly support a role for renal afferent signaling in sympathetic activation, but its quantitative contribution relative to RAAS activation, volume expansion, and metabolic derangements in human CKD has not yet been defined in adequately powered prospective interventional studies.

*Evidence summary:* Human studies support sympathetic activation in CKD, whereas the specific role of renal afferent signaling remains supported mainly by preclinical and translational evidence.

### 5.2 Uremic milieu, inflammation, and chemoreflexes

Human CKD studies demonstrate that uremic toxins, metabolic acidosis, and chronic inflammation in CKD modulate central autonomic circuits and peripheral chemoreceptors (Zoccali et al., 2025). Clinical observations in CKD patients demonstrate enhanced carotid chemoreflex sensitivity and sleep-disordered breathing in CKD further accentuate sympathetic surges, particularly at night (Beecroft et al., 2006; Mansukhani et al., 2014). Experimental models including LPK rats show systemic inflammation and oxidative stress, highlighted as central themes in the “behind the scene” review (Ameer, 2022), impair baroreflex function and endothelial NO signaling, compounding autonomic and vascular dysfunction (Figures 3, 5). These human and preclinical findings converge to generate a milieu where modest hemodynamic stress leads to exaggerated autonomic responses and sustained vasoconstriction. However, most human evidence is associative, and prospective studies disentangling the independent effects of uremic toxins, inflammation, and chemoreflexes from BP and volume status are still lacking.

*Evidence summary:* Uremia, inflammation, and chemoreflex sensitization are associated with autonomic dysregulation in CKD, but most human evidence remains associative.

## 6 Vascular consequences in chronic kidney disease associated hypertension

Multiple factors contribute to BP elevation, whether in essential hypertensive state, or CKD, or with the presence of both conditions that are often superimposed by autonomic burden (Table 2). In patients without CKD, endothelial dysfunction, increased PWV, and modest reductions in flow-mediated dilation are typically proportional to BP level and disease duration (Serhiyenko et al., 2023). By contrast, CKD-associated hypertension is characterized by disproportionately severe impairment of flow-mediated dilation, “excess” arterial stiffness at any given office BP, higher central systolic pressure, and more pronounced microvascular rarefaction, reflecting the combined impact of sympathetic overactivity, RAAS activation, uremic toxins, and oxidative stress on the vascular wall (Ameer et al., 2014; Seravalle et al., 2021; Ameer, 2022). Mediators like elevated endothelin-1 and reduced NO metabolites further shift the balance toward vasoconstriction, while abnormal ambulatory patterns with non-dipping or reverse-dipping and a high nocturnal load indicate impaired baroreflex and chemoreflex control, particularly during sleep (Sinha and Agarwal, 2015; Roumeliotis et al., 2020; Ameer, 2022; Grassi et al., 2022). Together, these markers support the concept that CKD-associated hypertension represents a state of amplified autonomic–vascular injury rather than simply “more severe” essential hypertension (Bidani et al., 2013; Ameer, 2022; Grassi et al., 2022). While these autonomic–vascular changes are consistent and biologically plausible, most available studies do not fully disentangle their independent effects from those of BP level, duration of hypertension, mineral bone disorder, and traditional cardiovascular risk factors.

### 6.1 Endothelial dysfunction and nitric oxide imbalance (evidence: level 2 + 3)

Human clinical studies confirm endothelial dysfunction is a hallmark of CKD and contributes to both the development and maintenance of hypertension. In CKD patients, uremia, oxidative stress, and chronic SNS overactivity reduce NO production and increase its degradation, shifting the balance toward vasoconstrictor mediators such as endothelin-1 (Figure 4A) (Daenen et al., 2019; Six et al., 2020; Ameer, 2022; Schiffrin and Pollock, 2024). CKD rodent models demonstrate sympathetic stimulation also promotes endothelial expression of adhesion molecules and pro-inflammatory cytokines, facilitating vascular inflammation and atherogenesis (Liang et al., 2013; Kaur et al., 2017). These convergent human and preclinical observations show the combination of impaired vasodilatory capacity and heightened vasoconstrictor responsiveness underlies the increased peripheral resistance typical of CKD-associated hypertension (Ameer, 2022; Liang et al., 2025).

*Evidence summary:* Endothelial dysfunction, reduced NO bioavailability, and arterial stiffness are consistently associated with autonomic imbalance in CKD, while direct causal links remain largely mechanistic.

### 6.2 Structural remodeling and stiffness

Human and animal CKD studies demonstrate that CKD accelerates arterial calcification, medial thickening, and fibrosis, driven by mineral bone disorder, inflammation, and neurohumoral activation (Ameer et al., 2015; Waziri et al., 2019; Kim and Hwang, 2021). LPK rat models confirm that SNS overactivity and RAAS activation stimulate smooth muscle proliferation and collagen deposition, contributing to arterial stiffness and PP (Figure 4B) (Ng et al., 2011; Ameer et al., 2014; Quek et al., 2016). These combined clinical and preclinical observations show this stiffening amplifies systolic pressure, widens PP (Figure 4C), and increases left ventricular afterload, promoting hypertrophy and heart failure. In parallel, clinical evidence confirms microvascular rarefaction, a loss of small vessels, which consequently reduces perfusion reserve and further elevates vascular resistance (Querfeld et al., 2020).

*Evidence summary:* Human data support increased arterial stiffness and microvascular rarefaction in CKD-associated hypertension, but the cellular pathways linking autonomic overactivity to remodeling are mainly experimental.

## 7 Integrated view: autonomic–vascular crosstalk in chronic kidney disease

### 7.1 Interactions with RAAS, sodium balance, and volume

Prior work highlighted salt and volume expansion, SNS hyperactivity, and upregulated RAAS as central, mutually reinforcing drivers of hypertension. Volume overload increases cardiac output and arterial pressure, which should enhance baroreflex-mediated sympathoinhibition, but in CKD the baroreflex is blunted and baroreceptors may reset to higher pressure levels (Figure 5) (Mancia and Grassi, 2014; Salman et al., 2015a; Ameer, 2022).

Angiotensin II and aldosterone stimulate central sympathetic nuclei and potentiate vasoconstrictor responses, while sympathetic activation enhances renin release, sustaining RAAS activity. Sodium retention and vascular stiffness further augment central sympathetic outflow and impair pressure natriuresis, creating a self-perpetuating cycle (Ameer, 2022; Maranduca et al., 2023).

In this framework, autonomic dysregulation is best viewed as an amplifier and integrator of other pathogenic pathways—rather than a stand-alone or exclusive driver—within the complex pathophysiology of hypertension in CKD.

*Evidence summary:* RAAS activation, sodium retention, and impaired baroreflex buffering are well established in CKD, but their relative contribution to autonomic dysregulation varies across patients and remains difficult to separate clinically.

### 7.2 Oxidative stress and endothelial–autonomic coupling

Oxidative stress, accentuated in CKD, reduces NO bioavailability and uncouples endothelial NO synthase, directly impairing vasodilation and baroreflex function. Reactive oxygen species in central autonomic regions also facilitate sympatho-excitation, with effects at both vascular and neural levels (Hirooka, 2011; Mancia and Grassi, 2014; Kaur et al., 2017; Ameer, 2022; Sabino-Carvalho et al., 2024).

Endothelial dysfunction impairs the normal buffering role of vasodilators on sympathetic vasoconstriction, making the vasculature more sensitive to catecholamines. This endothelial–autonomic uncoupling represents a critical pivot in the progression from adaptive responses to sustained, maladaptive hypertension (Figure 5) (Sprick et al., 2019; Cao et al., 2020; Roumeliotis et al., 2020; Ameer, 2022).

*Evidence summary:* Oxidative stress and endothelial dysfunction are closely linked to autonomic imbalance in CKD, but the extent to which they are primary drivers versus amplifiers of disease remains uncertain.

## 8 Clinical evidence of autonomic dysfunction in chronic kidney disease

Clinical evidence for autonomic dysfunction is common across the CKD spectrum and closely linked to BP patterns, vascular injury, and hard clinical outcomes (Table 3). In non-dialysis CKD stages 3–5, multiple cohorts have shown reduced global HRV, lower high-frequency power, and impaired BRS compared with matched controls; these abnormalities correlate with higher office and ambulatory BP, greater BPV, increased carotid–femoral PWV, and a higher incidence of left ventricular hypertrophy (LVH) and composite cardiovascular events during follow-up (Salman, 2015; Sinha and Agarwal, 2015; Lai et al., 2020; Serhiyenko et al., 2023). In maintenance hemodialysis patients, HRV and baroreflex indices are often profoundly depressed, with only transient improvement during or immediately after dialysis sessions; low standard deviation of normal-to-normal intervals (SDNN) and HF power are associated with non-dipping or reverse-dipping nocturnal BP patterns, increased arterial stiffness, and a substantially elevated risk of sudden cardiac death and all-cause mortality (Kuo et al., 2018; Osataphan et al., 2023). Studies comparing CKD stages 3–4 with and without diabetes report that CKD per se is associated with reduced HRV, while concomitant diabetes is linked to more severe cardiovascular autonomic neuropathy and orthostatic hypotension, which correlate with higher nighttime BP load, impaired dipping, and increased rates of cardiovascular hospitalizations (Tahrani et al., 2014). Microneurography sub-studies in CKD stages 2–4 demonstrate higher MSNA and lower HRV than in healthy controls, with elevated MSNA associating with reduced systolic dipping, higher nocturnal BP, impaired endothelial function, and greater arterial stiffness, predicting a higher cardiovascular risk profile (Kaur et al., 2017; Grassi et al., 2021). Finally, CKD cohorts that combine autonomic and inflammatory profiling show that low HRV and impaired BRS cluster with increased inflammatory and oxidative stress markers; patients with this “autonomic–inflammatory” phenotype have higher central BP and PWV and exhibit faster CKD progression and more cardiovascular events (Zoccali et al., 2025).

Importantly, while autonomic indices in CKD are consistently associated with vascular injury and adverse events across multiple cohorts, few studies have prospectively tested whether modifying these indices independently—beyond standard BP and risk factor control—improves hard clinical outcome (Table 3).

*Evidence summary:* Human studies consistently show that autonomic dysfunction is common in CKD and associated with vascular injury and adverse outcomes, but whether autonomic-guided treatment improves hard endpoints remains unproven.

### 8.1 Heart rate variability and baroreflex indices

Multiple studies document reduced HRV and impaired BRS in patients with moderate to advanced CKD and in dialysis populations, independent of traditional cardiovascular risk factors. Decrements in HRV and BRS correlate with higher ambulatory BP, greater arterial stiffness, and increased risk of sudden cardiac death (Sinha and Agarwal, 2015; Ameer, 2022; KenKnight and Verrier, 2022; Serhiyenko et al., 2023). These autonomic indices often worsen with CKD progression and may partially improve with nephron-sparing interventions, optimized volume management, or successful kidney transplantation. Such observations support the concept that autonomic imbalance is modifiable and clinically meaningful in this population (Rubinger et al., 2009; Jha et al., 2021).

### 8.2 Direct sympathetic measurements and neurohumoral markers

Studies employing radiotracer techniques and microneurography have revealed heightened renal and MSNA in CKD patients compared with matched controls. Plasma NE levels and other sympathetic biomarkers tend to be elevated and associate with LVH, arrhythmias, and adverse outcomes (Schlaich, 2011; Mancia and Grassi, 2014; Grassi et al., 2021). Neurohumoral profiling also shows increased renin and angiotensin II activity, particularly in proteinuric and volume-overloaded patients, which aligns with the integrative pathophysiologic model. These findings collectively position autonomic activation as a central feature of the CKD-hypertension axis (Figure 5) (Grassi et al., 2015; Guyenet et al., 2020; Ameer, 2022).

### 8.3 Direct human studies of autonomic-vascular dysfunction in CKD (evidence: level 1 + 2)

Direct human investigations validate autonomic-vascular mechanisms across CKD stages 2–5 (Table 2). Microneurography demonstrates elevated MSNA correlating with reduced eGFR, increased PWV, and impaired flow-mediated dilation (FMD) in CKD stages 2–4 (Grassi et al., 2022). Volume expansion studies confirm extracellular fluid overload sustains MSNA elevation in pre-dialysis CKD (Converse et al., 1992).

Recent clinical studies characterize impaired BP regulation in CKD stages 3–4. Blunted cardiac baroreflex sensitivity (cBRS) associates with increased beat-to-beat BPV despite similar mean BP, predicting non-dipping patterns (Sabino-Carvalho et al., 2023). Sympathetic baroreflex sensitivity (sBRS) is attenuated while sympathetic-BP transduction is reduced, challenging enhanced α1-adrenergic sensitivity assumptions and suggesting counter-regulatory vascular desensitization (Sabino-Carvalho et al., 2024).

Longitudinal data confirm clinical relevance. Reduced HRV predicts cardiovascular events and CKD progression in stages 3–5 (Chandra et al., 2011). Therapeutic interventions demonstrate reversibility: aerobic exercise prevents progressive MSNA and augmentation index increases over 12 weeks in CKD stages 3–4 (Jeong et al., 2023).

These key human studies provide direct translational evidence for autonomic-vascular mechanisms and suggest autonomic profiling as a potential biomarker for CKD hypertension management, though clinical thresholds and prognostic utility require prospective validation (Table 2).

*Evidence summary:* Direct human studies validate autonomic–vascular abnormalities across CKD stages, but clinical thresholds and prognostic utility still require prospective validation.

## 9 Therapeutic modulation of autonomic–vascular pathways

Table 4 outlines how different pharmacologic and device-based strategies target the autonomic axis in patients with hypertension and CKD, and how these translate into hemodynamic and vascular effects.

### 9.1 Established therapies with autonomic effects (evidence: level 1 + 2)

RAAS blockade and SGLT2 inhibitors—foundational CKD hypertension treatments—indirectly improve autonomic balance by attenuating angiotensin II-mediated sympathoexcitation, optimizing volume control, and enhancing endothelial function, with proven cardiovascular/renal outcome benefits (Eijkelkamp et al., 2007; Heerspink et al., 2020; Neuen et al., 2021; Zoccali et al., 2025).

### 9.2 Emerging/investigational autonomic strategies (evidence: level 2 + 3)

Renal denervation, baroreflex activation therapy, and vagus nerve stimulation reduce sympathetic markers in resistant hypertension (Esler et al., 2012; Mahfoud et al., 2022), but CKD-specific outcome data remain limited to small, selected series. Exercise rehabilitation shows HRV/BRS improvements in pilot CKD studies (Jeong et al., 2023) but lacks large-scale validation.

*Evidence summary:* Pharmacologic and device-based therapies can improve BP and some autonomic or vascular indices, but evidence that they reverse autonomic-driven vascular remodeling in CKD remains limited.

### 9.3 Pharmacological strategies

Pharmacological agents targeting autonomic-vascular mechanisms demonstrate translational efficacy from CKD rat models to human CKD cohorts (Pugh et al., 2019). Several established antihypertensive drug classes exert important autonomic and vascular effects beyond BP-lowering. The successful RAAS inhibitors reduce angiotensin II–mediated central sympathetic drive, improve endothelial function, and attenuate vascular remodeling, particularly relevant in CKD patients (Brenner et al., 2001; Lewis et al., 2001) and experimental models with proteinuria (Ameer et al., 2016; Ameer, 2022; Zoccali et al., 2025). Beta-blockers mitigate sympathetic cardiac and, to some extent, vascular effects, and are especially valuable in patients with coronary artery disease or heart failure, although their impact on arterial stiffness is variable across human cohorts (Eguchi, 2016; Joseph et al., 2019). Calcium channel blockers and diuretics primarily modulate vascular resistance and volume, but may secondarily influence autonomic tone by altering baroreceptor loading conditions in clinical CKD practice with preclinical parallels (Mancia and Grassi, 2014; Quek et al., 2020; Ameer, 2022; Zoccali et al., 2025). The relatively new (sodium-glucose co-transporter-2) SGLT2 inhibitors, though primarily renal and metabolic agents, improve volume control and may reduce sympathetic activity via nephron and tubuloglomerular mechanisms observed in human trials (Perkovic et al., 2019; Heerspink et al., 2020), with emerging data suggesting favorable cardiovascular effects in CKD mirroring LPK rat findings (Jeewandara et al., 2015). Mineralocorticoid receptor antagonists (MRA) also exhibit sympatholytic and vascular anti-fibrotic actions across species but require careful potassium monitoring in CKD patients (Baran et al., 2021; Kolkhof et al., 2022).

### 9.4 Device-based and neuromodulatory approaches

Device-based interventions targeting the autonomic nervous system are of growing interest in resistant hypertension and may have particular relevance to CKD. For example, renal denervation interrupts both efferent and afferent renal nerves, resulting in lowering sympathetic drive, reducing renin release, and decreasing vascular resistance (Mancia and Grassi, 2014; Wilson et al., 2020) but CKD-specific outcome data remain limited. While BAT involves chronic stimulation of carotid baroreceptors to enhance baroreflex-mediated sympathoinhibition (Victor, 2015), with evidence of BP reduction and improved autonomic indices in resistant hypertension (CKD applicability extrapolated) (Hering et al., 2013; Wallbach et al., 2016). Vagus nerve stimulation, extensively studied in heart failure, improves autonomic balance, HRV, and inflammatory markers, and could be explored in CKD cohorts with concomitant heart failure and high sympathetic tone (CKD-specific efficacy unproven) (Mancia and Grassi, 2014; Konstam et al., 2019; Gentile et al., 2024).

## 10 Future directions and research gaps

Future research must bridge human CKD cohorts with CKD rat-validated autonomic-vascular mechanisms to address critical translational gaps. There is a need for mechanistic and interventional studies specifically designed in CKD populations to disentangle the relative contributions of renal afferent signaling, RAAS, inflammation, and metabolic disturbances to autonomic dysregulation. Prospective human cohorts should integrate non-invasive autonomic biomarkers, such as HRV, BRS, and sympathetic imaging, should be integrated into longitudinal CKD cohorts to clarify prognostic significance and therapeutic responsiveness. However, standardized cut-offs for clinical decision-making, the incremental prognostic value of these indices beyond established risk markers, and their cost-effectiveness in routine CKD care remain to be defined.

Research should explore emerging research themes in autonomic and vascular regulation in CKD and hypertension. This includes mechanistic studies integrating autonomic phenotyping with advanced vascular imaging and interventional trials targeting autonomic pathways. Studies should focus on autonomic regulation of cardiovascular function and highlight opportunities for cross-disciplinary collaborations between nephrology, cardiology, and autonomic neuroscience. Personalized treatment strategies incorporating autonomic profiling could help identify patients who would benefit most from neuromodulatory therapies in addition to optimized pharmacologic and lifestyle measures.

Despite substantial mechanistic and clinical data, several pivotal questions remain about autonomic–vascular interactions in hypertension and CKD, as shown in Table 5. Cross-sectional and small longitudinal studies propose that autonomic dysfunction appears early in the CKD course, but its onset, trajectory, and dependence on underlying etiology are still poorly defined, necessitating large, stage-spanning cohorts with repeated HRV, BRS, and MSNA measurements. Existing works demonstrate that reduced HRV, impaired baroreflex function, and elevated sympathetic activity correlate with vascular damage and adverse events, yet their comparative and incremental prognostic value over standard risk markers has not been systematically established, requiring prospective CKD hypertension cohorts with harmonized autonomic and vascular phenotyping. Similarly, device-based neuromodulation (Victor, 2015), combined pharmacologic targeting of SNS, RAAS, and inflammation, and the practical integration of autonomic profiling into everyday nephrology practice have been only sparsely studied in CKD populations; carefully designed randomized trials, factorial intervention studies, and implementation research will be essential to determine whether these strategies can meaningfully reverse established vascular injury and enable more personalized risk stratification and treatment.

**Table 5:** Key unanswered questions on autonomic–vascular regulation in hypertension and CKD.

Research question	Rationale	Suggested study design/approach
How early does autonomic dysfunction appear across CKD stages and etiologies?	Cross-sectional data suggest autonomic abnormalities even in non-dialysis CKD, but timing, severity, and variation by primary kidney disease are not well defined.	Large multicenter cohorts with CKD stages 1–5, repeated HRV/BRS and MSNA (in subsets), stratified by etiology and comorbidities.
Which autonomic markers best predict vascular damage and cardiovascular outcomes in CKD-associated hypertension?	Reduced HRV, impaired BRS, and elevated MSNA each correlate with events, but comparative prognostic value and additive information beyond standard risk markers remain unclear.	Prospective CKD hypertension cohorts with systematic autonomic testing, vascular phenotyping (PWV, flow-mediated dilation, central BP), and hard outcome follow-up.
What is the incremental benefit of device-based neuromodulation (renal denervation, baroreflex activation, vagus nerve stimulation) in CKD populations?	Most neuromodulation trials under-represent CKD, and effects on renal function, vascular stiffness, and arrhythmic risk in this group are insufficiently studied.	Dedicated randomized trials or prespecified CKD sub-studies assessing BP, autonomic indices, vascular markers, renal endpoints, and safety.
Can combined targeting of SNS, RAAS, and inflammation reverse established vascular damage in CKD-associated hypertension?	Autonomic overactivity, RAAS activation, and inflammation act synergistically, but the reversibility of vascular remodeling with multi-target therapy is uncertain.	Factorial trials or stepwise intervention studies (e.g., RAAS blockade + SGLT2 inhibitor ± device-based therapy) with serial imaging of vascular structure and stiffness.
How can autonomic profiling be integrated into routine risk stratification and personalized treatment in CKD hypertension?	Autonomic tests are not routinely used in nephrology practice, and thresholds for intervention or treatment selection are undefined.	Implementation studies testing algorithms that incorporate HRV/BRS or simple reflex tests into decision pathways, with evaluation of feasibility, cost, and clinical impact.

Key Unanswered Questions on Autonomic–Vascular Regulation in Hypertension and CKD (Evidence Gaps: Level 1 + 2). Research agenda highlighting priority gaps in human clinical evidence (Level 1 + 2) requiring prospective studies to establish autonomic dysfunction timing, prognostic utility, neuromodulation efficacy, vascular reversibility, and clinical implementation strategies in CKD-associated hypertension. Table identifies evidence gaps addressed by Level 1 (direct autonomic measures) and Level 2 (vascular correlates) studies needed to advance from associative findings to causal, prognostic, and therapeutic understanding. See Methods Section 2 for evidence hierarchy details. CKD, chronic kidney disease; HRV, heart rate variability; BRS, baroreflex sensitivity; MSNA, muscle sympathetic nerve activity; PWV, pulse wave velocity; BP, blood pressure; SNS, sympathetic nervous system; RAAS, renin–angiotensin–aldosterone system; SGLT2, sodium–glucose co-transporter.

*Evidence summary:* Current human evidence supports autonomic dysfunction as a clinically relevant risk marker in CKD-associated hypertension, but autonomic-guided treatment strategies still require prospective testing.

## 11 Conclusion and clinical implications

This narrative review synthesizes robust Level 1 + 2 human evidence establishing sympathetic overactivity, impaired BRS, and reduced HRV as characteristic features of both essential hypertension and CKD-associated hypertension, with exaggerated severity and earlier onset in CKD stages 2–5. These autonomic abnormalities consistently correlate with vascular hallmarks including endothelial dysfunction, increased PWV, non-dipping BP patterns, and LVH, supporting the concept of CKD hypertension as an amplified neurovascular phenotype rather than simply more severe essential hypertension. The established therapies RAAS blockade and SGLT2 inhibition demonstrate indirect autonomic benefits alongside proven renal and cardiovascular outcome improvements, positioning them as cornerstones of current management. However, critical evidence gaps persist that limit translation to routine nephrology practice. Device-based neuromodulation strategies (renal denervation, BAT) and exercise-based autonomic rehabilitation show promise in surrogate endpoints but lack CKD-specific outcome data. Prospective studies are urgently needed to determine prognostic thresholds for autonomic markers, establish reversibility of vascular remodeling through multi-target approaches, and validate implementation strategies for autonomic profiling in risk stratification. Until these gaps are addressed, optimizing RAAS inhibition/SGLT2 therapy while monitoring for hyperkalemia, volume status, and treatment adherence represents the most evidence-based immediate approach to mitigating autonomic-vascular injury in CKD hypertension.
